# Effects of preferred music on physiological responses, perceived exertion, and anaerobic threshold determination in an incremental running test on both sexes

**DOI:** 10.1371/journal.pone.0237310

**Published:** 2020-08-12

**Authors:** Felipe Marroni Rasteiro, Leonardo Henrique Dalcheco Messias, Pedro Paulo Menezes Scariot, João Pedro Cruz, Rafael Lucas Cetein, Claudio Alexandre Gobatto, Fúlvia Barros Manchado-Gobatto

**Affiliations:** Laboratory of Applied Sport Physiology - LAFAE, School of Applied Sciences, University of Campinas, UNICAMP, Limeira, São Paulo, Brazil; Universidade Federal de Mato Grosso do Sul, BRAZIL

## Abstract

This study aimed to investigate and compare the effects of preferred music on anaerobic threshold determination in an incremental running test, as well the physiological responses and perceived exertion at this intensity, in physically active men and women. Additionally, by using area under the curve (AUC) analysis of the parameters of interest during the graded test, we studied the effects of music at two physiological moments—before and after anaerobic threshold intensity (iAT)—in men and women. Twenty (men = 10; women = 10) healthy and active participants completed four visits to the laboratory. The first and second sessions were used for sample characterization. In the third and fourth sessions, participants performed an incremental running test (started at 7 km.h^-1^ with increments of 1 km.h^-1^ at each 3-minute stage) under preferred music and non-music conditions. Blood lactate ([Lac]), heart rate (HR), and perceived exertion were measured by two scales (RPE_Borg_ and the estimation of time limit ‒ ETL) during all tests, and the total time of effort (TT) was considered as performance. Individual curves of the “*intensity vs blood lactate*” analyzed by the bissegmentation method provide the iAT and the AUC of [Lac], HR, RPE_Borg_, and ETL before and after the iAT attainment were calculated. The iAT for men (non-music: 11.5±0.9km.h^-1^ *vs* music: 11.6±1.1km.h^-1^) and women (non-music: 9.8±0.7km.h^-1^ *vs* music: 9.7±0.7km.h^-1^) was not affected by music, and for both sexes, there was no difference between non-music and music conditions in all variables obtained at iAT. The AUC of all variables were not affected by music before the iAT attainment. However, [Lac], HR, and RPE_Borg_ presented higher values of AUC after iAT for the female group with preferred music. This may be due to the fact that 70% of women have increased TT under music conditions. Overall, preferred music did not affect the iAT determination in an incremental running test. However, some physiological responses and perceived exertion after iAT of female subjects seems to be influenced by preferred music.

## Introduction

The effect of music on exercise was investigated by Ayres [1], who described the influence of a music band playing during a six-day bicycle race. However, only in recent decades has there been a significant increase in research on the importance of this topic to the performance in different physical activities and exercise situations [2–8]. Many investigations involving the effect of music on exercise have been focused on some contributing factors of music, such as genre [9], rhythm [10], tempo [11, 12], and auditory–motor synchronization [5, 8]. On the other hand, there are still few studies in which the musical preference of the participants is freely guaranteed. It has been speculated that emotional components of the preferred music could be an effective aid to increase personal motivation [13–15].

The literature presents conflicting information regarding the influence of music (preferred or not) on exercise at different intensity domains [16–19]. Based on evidence that music can distract exercisers from the unpleasant and uncomfortable sensations associated with physical effort [8, 20], it should be expected that preferred music would help not only in exercise at moderate or heavy domains but especially at severe domain, in which physiological instability and exhaustion meet. Although researchers have concerned with distinct exercise characteristics in experiments with music (e.g. type, intensity and volume) [21–24], few investigations considered this context regarding the preferred music [16, 17]. To the best of our knowledge, despite the significance of the anaerobic threshold intensity (iAT) determination to define the aerobic–anaerobic transition and thus to obtain an accurate performance diagnosis on the exercise domains [25, 26], none investigations followed this way using the preferred music.

Among many evaluative protocols, the incremental (graded test) is the most common procedure for iAT determination in laboratory and field conditions [27]. During this application, the intensity is incremented, inducing an exponential behavior of blood lactate ([Lac]) accumulation. Therefore, the highest workload that still leads to an equilibrium between lactate production and removal is termed as the iAT [25] and can be determined by reliable mathematical analysis, such as the bissegmentation of two linear regressions along with linear interpolation [28–30]. Before the iAT attainment (moderate and heavy domains), physiological responses are not expected to abruptly increase, reflecting a stability that is favorable to maintenance of exercise [31, 32]. On the other hand, at intensities higher than iAT (severe domain), the physiological balance is gradually lost, leading to exhaustion [33]. We believe that investigations on the physiological responses (e.g., heart rate and [Lac]) and perceived exertion (obtained by perception scales) at two moments during the incremental test (before and after iAT) can improve the understanding of the effects of music at these different intensity domains. For this, the area under the curve analysis (AUC) commonly used in other scientific approaches [34, 35] seems to be an interesting method.

Although there are studies documenting the effects of music on exercise in both men and women [5, 11, 12, 36–40], there is still an ongoing debate about sex differences in music processing. It is reasonable to consider that music’s effects on physical performance could be sex-dependent, as there are reports showing that men and women seem to differ in their perception of music [41–45]. In this way, Macone et al. [39] and Cole and Maeda [36] have demonstrated that women, but not men, had increased physical performance in a music condition compared to a non-music condition. Thus, an important question to be answered is whether music affects differently the physiological responses, perceived exertion, and, consequently, the iAT determination in men and women in the same physical condition (e.g., active individuals but non-athletes). Still, the understanding of the effects of music on men or women depending on the intensity domains (before or after iAT) seems to add knowledge for exercise prescription in these conditions. Obviously, in this sense of application, the music to be used needs to be the subject's preference.

This study aimed to investigate and compare the effects of preferred music on anaerobic threshold determination in an incremental running test, as well as the physiological responses and perceived exertion at this intensity, in physically active men and women. Additionally, by using AUC analysis of the parameters of interest during the graded test, we studied the effects of music at two physiological moments (before and after iAT) in men and women.

## Materials and methods

### Study design

Participants were requested to maintain the same individual hydration/food habits and avoid alcohol/caffeine ingestion as well as hard physical activity at least 96 hours prior to testing. Twenty healthy, non-athletes, non-smoking, and active male (n = 10; age = 23±2 years; body mass = 73.3±11.7 kg; height = 175±1 cm; body fat = 8.5±2.3%) and female (n = 10; age = 20±1 years; body mass = 59.7±5.3 kg; height = 165±1 cm; body fat = 18.4±3.6%) were selected for this study. As inclusion criteria, individuals should be active and experienced with at least two years of weekly practice in running exercise. The present study was conducted according to the norms of Helsinki and previously approved by the Research Ethics Committee of The School of Medical Sciences, located at the University of Campinas (protocol number ‒ 64648617600005404).

**Fig 1:**
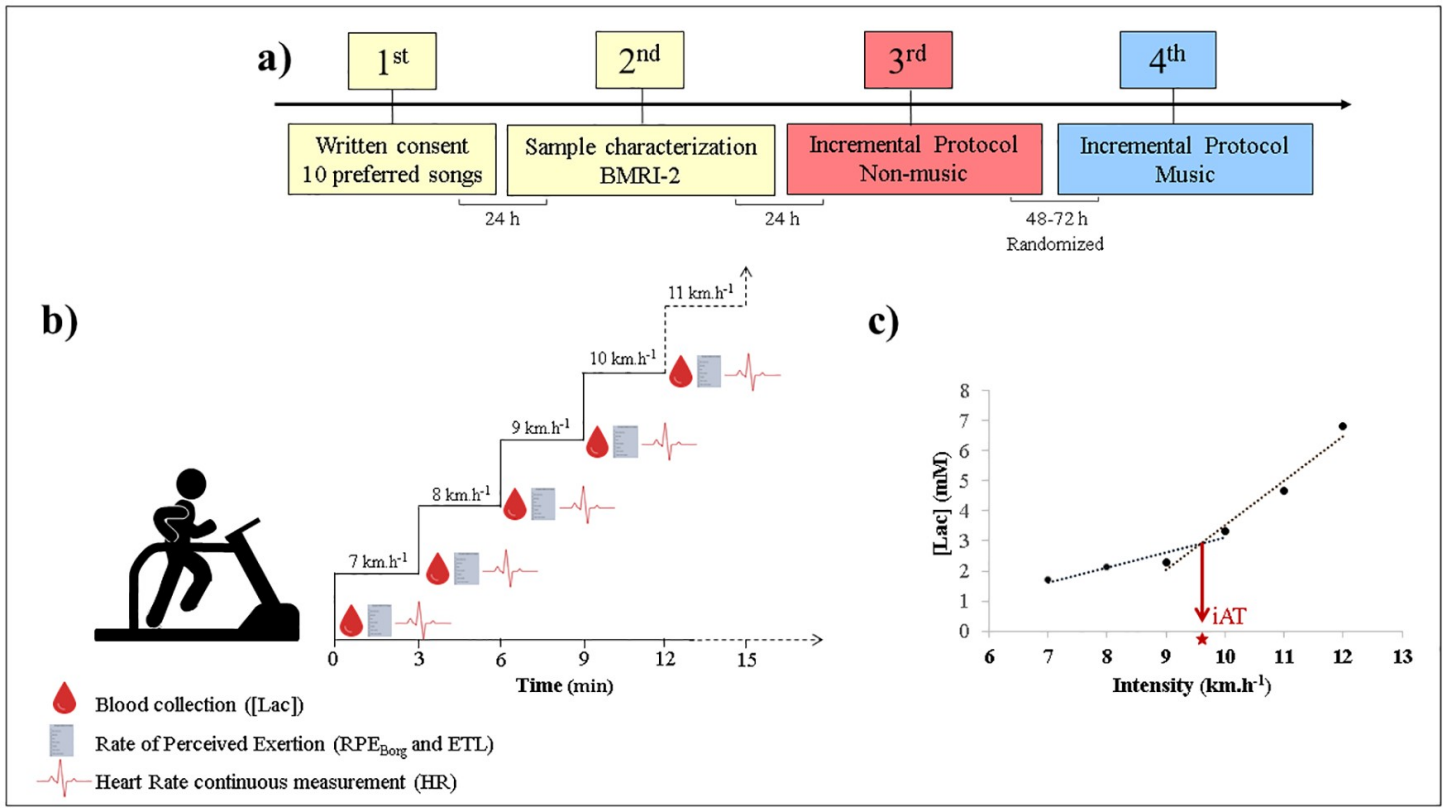
Experimental design adopted in the study. **a)** First session was conducted to explain the study’s procedures and obtain agreement to participate in the research. Moreover, subjects were instructed to bring 10 songs for exercising. Subsequently, during the second visit, subjects were evaluated for body composition, physical activity, and readiness. In the same session, the motivational quotient of the 10 songs provided by the subjects was determined via the Brunel Music Rating Inventory– 2 (BMRI-2). The incremental protocol in non-music and preferred music conditions was randomly conducted in the third and fourth sessions. **b)** Incremental protocol started at 7 km.h^-1^ with increments of 1 km.h^-1^ in 3-minute stages. Blood samples were collected at rest and at the end of each stage to [Lac] analysis. During the same interval, two perceived exertion scales were applied (RPE_Borg_ and ETL). HR was measured throughout the protocol. **c)** Anaerobic threshold intensity (iAT) was determined by the intersection between two linear fits resulting from the bissegmentation method.

Four visits to the laboratory were completed (Fig 1). The first session was conducted to explain the study’s procedures and obtain agreement to participate in the research, which was approved by the university’s local ethics committee. Moreover, at the end of this session, the subjects were asked to provide 10 songs they consider motivational during their daily physical activity. In line with this, the second session was conducted to identify the motivational level of each selected song via the Brunel Music Rating Inventory-2 [46]. During the same session, participants were evaluated for body composition (i.e., lean mass, fat mass, and body fat), physical activity level, and physical activity readiness (PAR-Q) [47]. Skinfold measurements were performed by the same experienced researcher via a clinical adipometer/plicometer (Cescorf, Cardiomed, PR, BR). Lohman [48] and Jackson and Pollock's [49] approaches were used to estimate the body composition of the men (i.e., triceps, subscapular, and abdominal skinfolds) and women (i.e., triceps, suprailiac, and thigh skinfolds), respectively. The International Physical Activity Questionnaire (IPAQ) [50] was adopted for analysis of physical activity level (men = 3535±2425 metabolic equivalent-min/week; women = 3568±1860 metabolic equivalent-min/week).

The third and fourth sessions were dedicated to the exhaustive incremental protocol on a motorized treadmill (Super ATL, Inbramed, RS, BR). All procedures were conducted in a controlled environment (temperature = 22°C±1°C; luminosity = ~300lx). Additionally, these sessions were conducted in an isolated room (length = 4.83 m; width = 2.11 m). Therefore, participants did not maintain contact with other people except for the evaluators, who communicated (when strictly necessary) through gestures. Moreover, information regarding the duration of the test or stages was avoided. These sessions were randomized and separated by 48‒72 hours (S1 File). In one of the sessions, the protocol was performed under non-music conditions. In the remaining session, subjects were allowed to listen to their preferred music during the exhaustive incremental protocol. In both evaluations, [Lac], HR, RPE_Borg_, ETL, and total time (TT) were analyzed.

### Music classification

The BMRI-2 evaluates the motivational quality of music during exercise. It is comprised of six items, each concerning a musical component (rhythm, style, melody, tempo, instrumentation, and beat). Each item is comprised of a seven-point Likert scale, where 1 is “strongly disagree” and 7 is “strongly agree" [46]. Taking into account hygiene and preference aspects, each individual was asked to bring his or her own headphones. For BMRI-2 application, the previously selected songs were inserted into a musical player (iPod Shuffle A1373, Apple, SP, BR), which was also used in the incremental protocol.

The music’s volume was standardized at five clicks below the maximum, ranging from 70‒85 dB. These strands were previously tested for the application of the BMRI-2, and it was found that the aforementioned range would be ideal for working within the present study precisely because it assures auditive safety. The instrument application consisted of the individual playing of the song for 90 seconds. Subsequently, the scale was applied and the song score established. In order to eliminate the effect of listening to the previous song, a concentration grid [51] was applied between songs. These processes were repeated until the establishment of the score referring to the 10 songs. During the incremental protocol, the songs were ranked according to the score previously obtained, with the highest score at the top of the playlist and the others placed in descending order (S2 File). Since all songs were considered preferred by the evaluated participants, the music tempo was not controlled. However, all bpm values are presented in the S3 File.

### Blood lactate concentration and heart rate analysis

Capillarized blood samples (25 μL) were taken from the earlobe and deposited into microtubes (Eppendorf 1.5 ml) containing 50 μl of NaF. The [Lac] was analyzed by the electrochemical method using a lactimeter YSI2300-STAT-Plus (Yellow Springs, OH, USA). The participants’ HR was recorded (beat to beat) using Polar heart monitors (Polar, RS800, RJ, BR). Data were recorded during all protocols.

### Perceived exertion scales

Two psychometric scales were considered for measurement of perceived exertion. The scale originally proposed by Borg [52] with a range of 6‒20 (RPE_Borg_) was adopted. Moreover, the estimation of time limit (ETL) scale proposed by Garcin et al. [53] was also considered.

### Incremental protocol

The incremental protocol started at 7 km.h^-1^, with increments of 1 km.h^-1^ in 3-minute stages. The slope of the treadmill was maintained at 1% during all tests. At the end of each stage, the effort was interrupted for 30 seconds for blood collection. During this interval, participants indicated with their fingers the perceived exertion in two psychometric scales. In both tests (non-music or music), the participants used the same auricular headsets adopted to answer the BMRI-2 in the second session.

For determination of the iAT, individual curves of intensity (km.h^-1^) *vs* blood lactate (mM) were plotted. After visual inspection, performed by two experienced researchers, the bissegmentation analysis proceeded and iAT was identified by the intersection between fits [28, 29]. [Lac], HR, RPE_Borg_, and ETL at iAT ([Lac]_iAT_, HR_iAT_, RPE_Borg iAT_, and ETL_iAT_, respectively) were determined by linear interpolation. Relativization in percentage (%) was performed by dividing the iAT by the maximum value recorded of intensity (i_peak_) and then multiplied by 100 (iAT [% i_peak_]). The same procedure was applied to calculate the [Lac]_iAT_ (% [Lac]_peak_) and HR_iAT_ (%HR_max_). TT was considered when the individual achieved maximum HR (i.e., 220-age) [54] or asked to stop (voluntary exhaustion). To calculate the time taken to reach the anaerobic threshold (TB_iAT_ [%TT]) as well as the remaining effort time (TA_iAT_ [%TT]), the intensity (km.h^-1^) and time (total seconds of each stage) were plotted as x axis and y axis, respectively. Thus, the first-degree equation was replaced by known values, identifying the specific time that the iAT occurred.

### Area under the curve analysis

**Fig 2:**
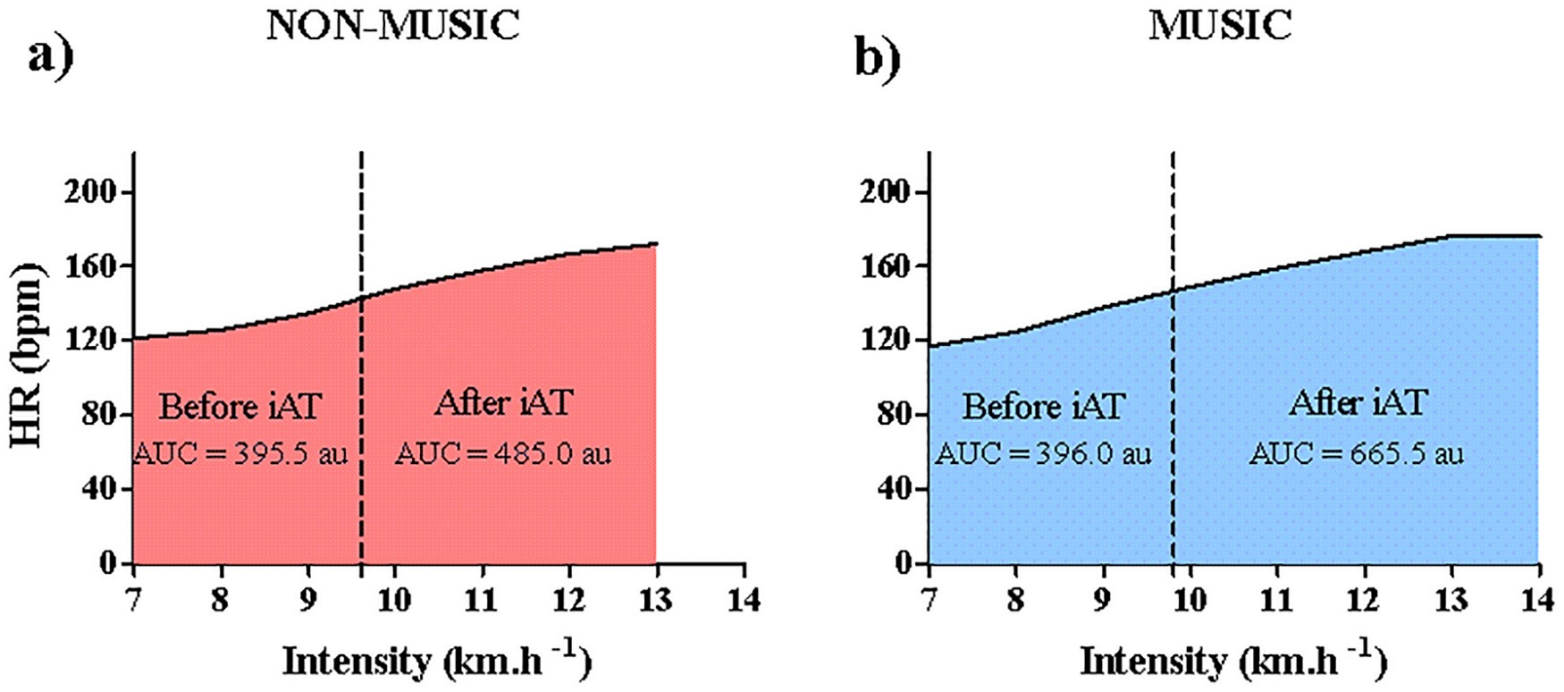
Individual example of the AUC analysis by Heart Rate (HR) responses. (**a**) Present the HR responses during the incremental running test under the non-music condition; (**b**) present the HR responses during the incremental running test under the preferred music condition. The **dotted line** indicates the anaerobic threshold intensity (iAT) in their respective conditions (non-music and music). Values represent the total area under the curve before and after the iAT. **au** indicates arbitrary unit.

Measurements obtained multiple times from the incremental protocol were also used to investigate whether music would be able to differently influence the responses before and after iAT attainment. Following the iAT determination, individual curves of intensity (km.h^-1^) *vs* the variables studied ([Lac], HR, RPE_Borg_, and ETL) were plotted. The curve was divided into two moments, before and after iAT. Then the trapezoidal method was applied stage by stage until reaching the stage corresponding to iAT. The AUC values obtained for each stage interval were then summed, and the total was considered as the AUC before iAT. The same was applied in the stages after the iAT. Fig 2 indicates an individual example of the AUC analysis of the heart rate variable.

### Statistical analysis

Data (S4 File) were calculated and analyzed using STATISTICA 7.0. The figures were elaborated by the software GraphPad Prism 5. Data are presented as mean and standard deviation of the mean. The normality and homogeneity of the data were confirmed by the Shapiro–Wilk and Levene tests, respectively. Two-way ANOVA was adopted to determine the effects of music (non-music *vs* music) and sex (male *vs* female), as well as their interaction (music *vs* sex) on parameters obtained from the incremental test. AUC data were analyzed by repeated measures ANOVA considering the effects of music (non-music *vs* music) and moment (before *vs* after iAT), as well as their interaction (music *vs* moment). The Newman–Keuls post hoc analysis was adopted in all cases. The relationship between variables was analyzed using Pearson’s correlation. In all cases, the level of significance was set at 5%.

## Results

**Fig 3:**
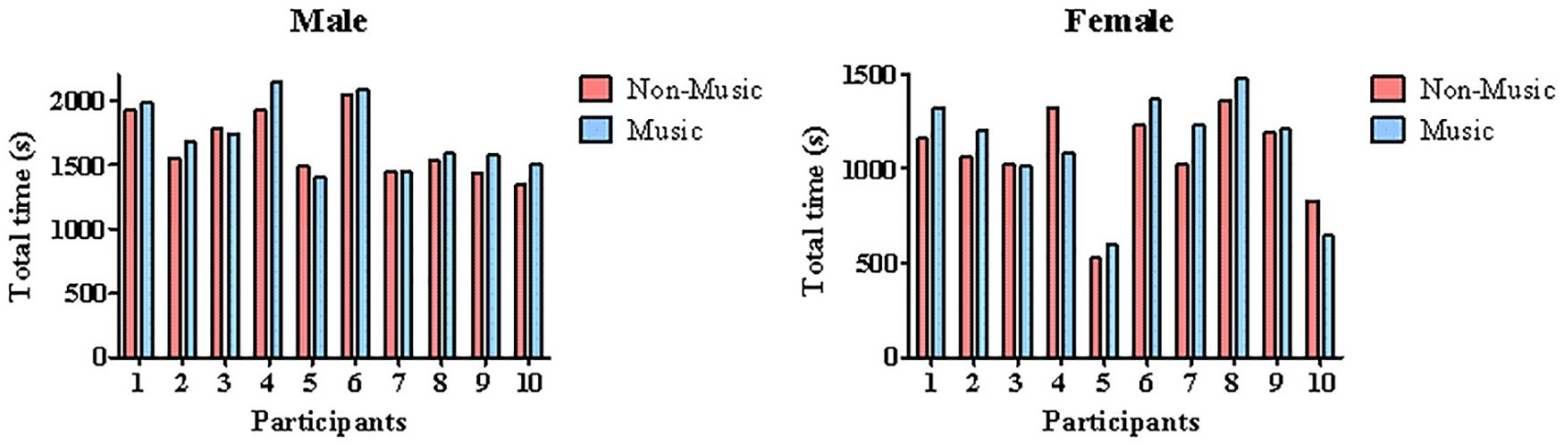
Individual results of the total time of effort (TT) obtained from the incremental protocol performed in non-music and music conditions.

Preferred music did not influence iAT determination through the incremental test, regardless of sex (Table 1). No significant effect of music on any of the variables studied was detected by two-way ANOVA. Additionally, we found an effect of sex on iAT and TT, showing that males exhibit higher aerobic fitness and physical performance (iAT and TT) than females. However, the female group presented higher HR_iAT_ values than the male group in the non-music condition, but no significance was observed for the music condition. No interaction effect was observed in any of the variables studied. In addition, most of the variables presented a significant relationship in the intra-group analysis under non-music and music conditions for the male group (**iAT**–r = 0.92, p = 0.001; **[Lac]**_**iAT**_−r = 0.79, p = 0.006; **[Lac]**_**iAT**_ **(% [Lac]**_**peak**_**)**–r = 0.80, p = 0.005; **TT**–r = 0.93, p = 0.001; **RPE**_**Borg iAT**_**−**r = 0.65, p = 0.042; **ETL**_**iAT**_−r = 0.91, p = 0.001). Likewise, **[Lac]**_**iAT**_ (r = 0.83, p = 0.003), **HR**_**iAT**_ (r = 0.98, p = 0.001), **HR**_**iAT**_ **(%HR**_**max**_**)** (r = 0.97, p = 0.001), **TT** (r = 0.86, p = 0.001), **RPE**_**Borg iAT**_ (r = 0.69, p = 0.028), and **ETL**_**iAT**_ (r = 0.87, p = 0.001) were significantly correlated for females. Individual responses regarding TT can be seen in Fig 3.

**Table 1:** Parameters obtained from the incremental protocol performed under non-music and music conditions, in both sexes.

	Male		Female		Music Effect		Sex Effect		Interaction	
	Non-music	Music	Non-music	Music	p	F	p	F	p	F
**iAT (km.h**^**-1**^**)**	11.5 ± 0.9	11.6 ± 1.1	9.8 ± 0.7^†^	9.7 ± 0.7^γ^	0.972	0.001	< 0.001	40.344	0.795	0.069
**iAT (% i**_**peak**_**)**	74.4 ± 3.0	73.3 ± 3.2	78.9 ± 5.4	77.1 ± 4.9	0.297	1.119	0.004	9.691	0.800	0.065
**[Lac]**_**iAT**_ **(mM)**	3.6 ± 1.0	3.6 ± 0.7	4.6 ± 2.4	5.1 ± 1.6	0.668	0.188	0.016	6.460	0.630	0.236
**[Lac]**_**iAT**_ **(% [Lac]**_**peak**_**)**	48.5 ± 5.0	45.5 ± 6.2	55.3 ± 13.5	53.0 ± 12.8	0.398	0.731	0.032	5.000	0.911	0.013
**HR**_**iAT**_ **(bpm)**	153 ± 10	152 ± 10	164 ± 13^†^	165 ± 13	0.971	0.001	0.002	11.279	0.829	0.047
**HR**_**iAT**_ **(%HR**_**max**_**)**	77.4 ± 4.8	77.1 ± 5.0	82.1 ± 6.2	82.5 ± 6.1	0.974	0.001	0.007	8.338	0.821	0.052
**TT (s)**	1644 ± 248	1710 ± 269	1073 ± 248^†^	1115 ± 293^γ^	0.525	0.413	< 0.001	48.395	0.887	0.021
**TB**_**iAT**_ **(%TT)**	60.3 ± 4.4	58.5 ± 5.1	61.1 ± 8.2	59.6 ± 5.6	0.382	0.782	0.618	0.254	0.940	0.006
**TA**_**iAT**_ **(%TT)**	39.7 ± 4.4	41.5 ± 5.1	38.9 ± 8.2	40.4 ± 5.6	0.382	0.782	0.618	0.254	0.940	0.006
**RPE**_**Borg iAT**_ **(score)**	13 ± 1	12 ± 1	13 ± 2	13 ± 1	0.488	0.492	0.041	4.498	0.299	1.113
**ETL**_**iAT**_ **(score)**	12 ± 3	11 ± 3	12 ± 3	12 ± 3	0.374	0.809	0.758	0.097	0.508	0.447

**iAT** − anaerobic threshold intensity; **iAT (% i**_**peak**_**)–**relativization of anaerobic threshold intensity in relation to the maximum intensity reached in protocol; [**Lac]**_**iAT**_−blood lactate concentration at iAT; **[Lac]**_**iAT**_ **(% [Lac]**_**peak**_**)** ‒ relativization of the lactacidemia referring to the iAT in relation to the lactate peak value obtained in the protocol; **HR**_**iAT**_−heart rate at iAT; **HR**_**iAT**_ **(%HR**_**max**_**)** − relativization of the heart rate referring to the iAT in relation to the product of the equation 220-age; **TT–**total time effort; **TB**_**iAT**_ **(%TT)** − relativization of the time to reach the iAT in relation to the total time of effort; **TA**_**iAT**_ **(%TT)** − relativization of the total time after reached the iAT in relation to the total time of effort; **RPE**_**Borg iAT**_**−**rating of perceived exertion at iAT; **ETL**_**iAT**_−estimation of time limit at iAT.

^γ^ significant difference between male and female in the preferred music condition.

^†^ significant difference between male and female in the non-music condition. Significance was pre-fixed at p ≤ 0.05.

**Fig 4:**
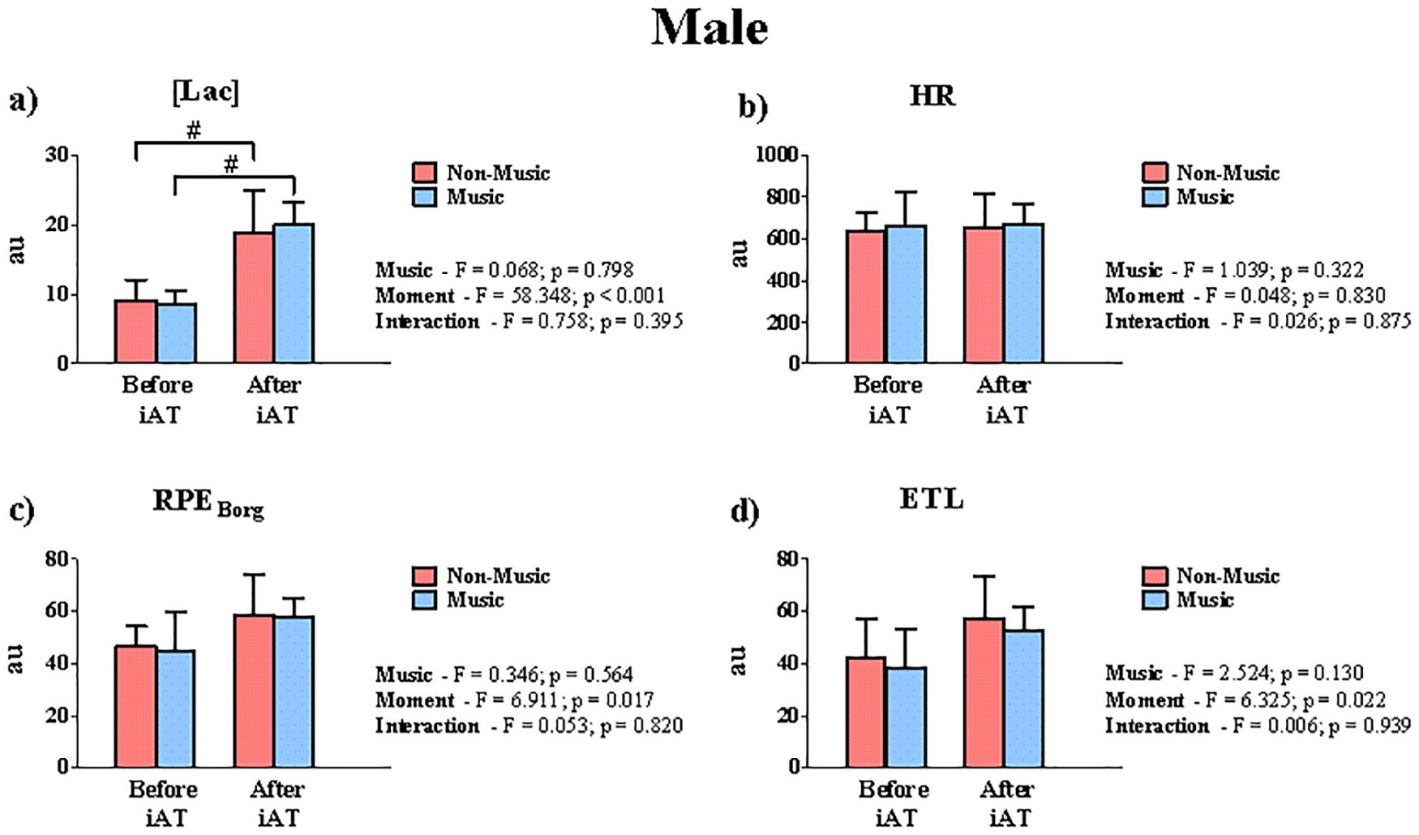
AUC analysis of male subjects on the [Lac], HR, RPE_Borg_, and ETL measured during the incremental test performed in music and non-music conditions. The AUC before and after the iAT in terms of (**a**) Lactate concentration [Lac], (**b**) Heart rate (HR), (**c**) Rate of perceived exertion (RPE_Borg_), and (**d**) Estimation of time limit (ETL) were compared. # indicates differences for the moment effect.

**Fig 5:**
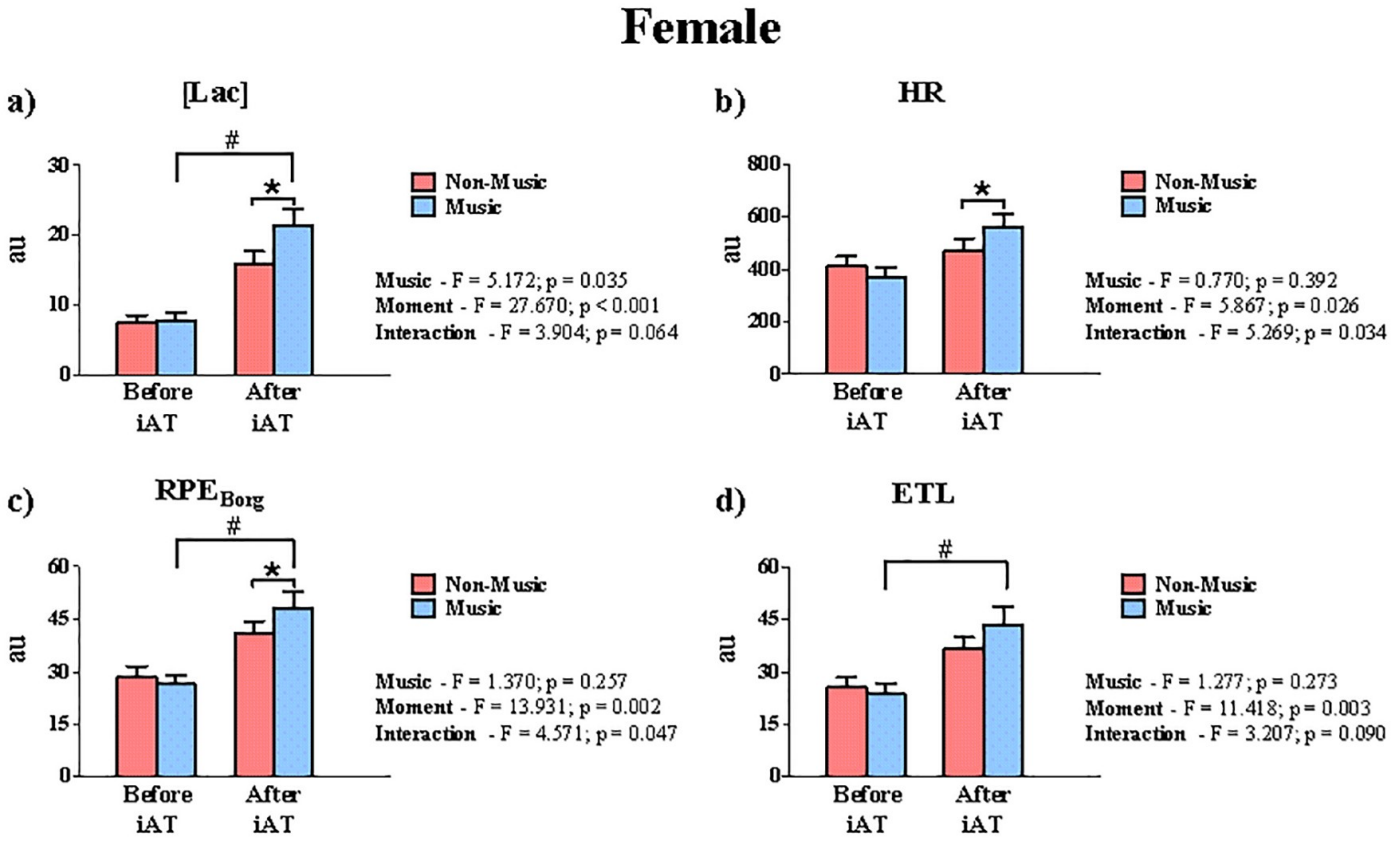
AUC analysis of female subjects on the [Lac], HR, RPE_Borg_, and ETL measured during the incremental test performed in music and non-music conditions. The AUC before and after the iAT in terms of (**a**) Lactate concentration [Lac], (**b**) Heart rate (HR), (**c**) Rate of perceived exertion (RPE_Borg_), and (**d**) Estimation of time limit (ETL) were compared. # indicates differences for the moment effect.

Figs 4 and 5 show a comparative analysis of the AUC from before and after the iAT attainment, under non-music and music conditions, for both sexes. For the male group, [Lac] presented a significant difference only for the moment effect (Fig 4a). On the contrary, HR did not present a significant difference when the moments were compared (before and after iAT). In the same way, RPE_Borg_ and ETL did not present any significance among the effects (music, moment, or interaction). On the other hand, [Lac] (Fig 5a), HR (Fig 5b), and RPE_Borg_ (Fig 5c) were significantly higher with the preferred music than non-music condition after iAT attainment for female subjects. The same was not observed for the ETL (Fig 5d).

## Discussion

Our main results demonstrate that in general terms, the preferred music did not significantly affect the physiological and perceptual responses during an incremental test, or the iAT determination. However, the significant effect for sex in terms of iAT and TT shows that men had higher aerobic fitness and performance in the incremental test when compared to women. Although we did not observe the effect of music on performance, independently of sex, intrasubject analysis revealed that most of the males evaluated (70%) had 2‒11% improvement in TT in the presence of preferred music. The same was observed for the females evaluated (70%), who had 2‒20% improvement in TT when the graded test was performed listening to preferred music. Moreover, AUC analysis revealed that [Lac] and perceived exertion (i.e., RPE_Borg_ and ETL) are elevated after iAT determination for both sexes (i.e., moment effect). On the other hand, women seem to be more susceptible than men to preferred music after iAT in terms of [Lac], HR, and RPE_Borg_, and this can partially explain the individual performance improvements. As far as we know, this study is the first to investigate the effect of preferred music in a running incremental test applied for male and female.

### Effects of sex and music on iAT determination and incremental test outcomes

Sexual dimorphisms and gender disparity in sports and exercise science have been highlighted [55]. Few evidences consistently demonstrate that men present higher performance than woman in incremental testing [56, 57]. A higher performance in men than women could be explained by differences in body composition components and their distribution. Men and women may differ in the amount and distribution of body fat [58] as well as lean body mass and body size like stature [59–61]. Hoffman et al. [62] showed that men have higher iAT than women in a cycle-ergometer. Moreover, only 11% of women maintained [Lac] in a steady state in exercise performed above iAT for 30 min. Estradiol may impact [Lac] dynamics in luteal and follicular menstrual phases [63], and Hoffman et al. [62] explained these marked oscillations in [Lac] are due to this ovarian hormone. However, a recent study came to the opposite conclusions, demonstrating that the power output and key physiological variables at maximal lactate steady state were not affected by the menstrual cycle [64]. On the other hand, it is important to state that besides [Lac]_iAT_, the HR_iAT_ and RPE_Borg iAT_ were also higher in women than men regardless of the adoption of music. We cannot affirm that our female data was affected by the menstrual cycle, but the early [Lac] increase may be associated with the circulating ovarian hormones, although this remains to be elucidated. Overall, although we cannot directly discuss the influence of hormonal status on the incremental test outcomes, we can affirm that, at least in our sample, the oxidative system of the female group was lower than in the male group, and this is not affected by preferred music.

Despite the comparisons between sexes had advanced on some scientific questions, the effects of music on the incremental test outcomes are a novel finding. To the best of our knowledge, a similar experimental design was not found, and our study provides new insights on this context. Music may affect the central nervous system by downregulating theta waves in brain regions during exercise [65]. Probably through these central mechanisms, the music seems to reduce the perceived exertion during exercise [8]. Other studies have also demonstrated that music can influence peripheral variables [66, 67]. In short, music may have an ergogenic effect on physical exercise [16, 68, 69]. We believe that our data offer two major insights on the music–exercise association. To begin with, music did not affect iAT and related parameters, regardless of sex. This important finding demonstrates the robustness of iAT determination.

Moreover, although ANOVA did not reveal any significant effect for music, 70% of the female group and 70% of the male group had 2‒20% and 2‒11% improvements in TT, respectively (Fig 3). Overall, our data suggest that women were more susceptible to music’s effects than men. In a mixed sample, Cole and Maeda [36] showed that only women had better performance in running while listening to preferred music. These authors suggested that women pay more attention to music while exercising than men, explaining the divergent outcomes. We must recognize that other music characteristics (e.g., synchronous, asynchronous) are also tested during exercise [2–5, 69–74] and may likely influence sex comparisons. The preferred characteristics were chosen for two main reasons. First, studies have demonstrated their ergogenic effect on exercise [16, 18, 75, 76], and this model matches our aims. Second, athletes and/or merely active subjects routinely use preferred music during exercise [8, 76–80]; therefore, our results have relevant practical applications. Although the analysis of preferred music on iAT determination and performance can reveal important outcomes, it does not allow further insights on the behavior of physiological variables and perceived exertion throughout the incremental test. Therefore, the AUC analysis supports this context.

### Physiological variables and perceived exertion before and after iAT

As far as intensity is incremented during the graded test, [Lac] is expected to abruptly increase when pyruvate oxidation exceeds its maximal rate of production. Therefore, higher [Lac] is expected after iAT attainment when compared to its counterpart. This is confirmed by our [Lac] AUC analysis for both groups (Figs 4a and 5a). However, preferred music may affect, only for the female group, AUC of [Lac] throughout the incremental test, after the iAT attainment. On the other hand, this result may be due to the increase of the TT by more than half of the female subjects. Studies analyzing the effects of music on [Lac] are scarce. Eliakim et al. [66] demonstrated that motivational music leads to higher lactate clearance after subjects performed a 6-min run exercise at peak aerobic power. This result was explained by the fact that music kept subjects active after exercise, promoting lactate clearance. This context, however, does not apply to our study, since we measure [Lac] during the incremental test. Although authors have showed that music can influence the central nervous system during exercise [7, 65], we cannot observe a direct relationship between preferred music and myocyte response in terms of lactate production. However, we observed a possible relationship between the preferred music and blood lactate response in the female group (Fig 5a), but further studies are required.

Music is capable of modifying the cardiovascular profile during exercise [81, 82]. Distinct from kinetic [Lac], HR increases linearly throughout the incremental test. The similarity of HR AUC between moments can be explained by a slight right-shift on the iAT determination for three subjects. This outcome reduced the AUC of these subjects after iAT attainment and explains the non-significant effect for moment (Fig 4b). The same results are not transposed to women. Music and moment were factors that modulated HR throughout the incremental testing. Since women tend to focus on some elements of music more than men [36, 43], it is possible that music increased the HR AUC of women mainly after iAT determination. Moreover, this partially explains why 70% of women had better performance (i.e., TT) in the incremental test with preferred music.

The effect of music on perceived exertion during exercise is one of the most discussed [8, 16, 18, 19, 67, 75, 78]. Nakamura et al. [16] showed that preferred music increases cycling distance performed at high intensity. Supported by the psychobiological model, Marcora et al. [83] suggest that exercise tolerance increases by the potential motivation of preferred music; others have supported this hypothesis [19, 77, 78, 81]. Thus, the significant interaction for RPE_Borg_ (Fig 5c) can be explained by the fact that preferred music improved exercise tolerance (TT), leading female subjects to present higher values of AUC. These inferences, however, are aligned only regarding our female subjects, and the same explanation in terms of TT and HR differences for both sexes fits in this case. Lastly, the ETL has been considered an important exercise context [84–86]. However, we do not know to what extent the complexity in estimate exercise duration is affected by music. Thus, our data cannot confirm that ETL is not sensitive to music effects, so further studies are required.

Finally, some studies have highlighted the importance of the music tempo on the running cadence [21, 23, 24, 87], but this effect was not considered over the preferred song. Interestingly, Dyer and McKune [88] investigated the tempo of individual favorite song on the performance, psychological and physiological responses of well-trained cyclists in time trial cycling. For a better investigation of the preferred music, the authors modified the music tempo according to three experimental conditions (100, 120 and 140 bpm). The authors observed a negative effect of the fast music tempo (i.e., 140 bpm) on the performance. Although they used a creative alternative to investigate the music tempo during the evaluation, the preferred characteristics of the song (for example, style, rhythm and harmony) had to be changed [89], possibly generating a different condition of that aimed in our study. For this reason, our group chose to evaluate the “pure effect” of the preferred music (without manipulating any property of music) in an incremental running test with controlled exercise cadence.

### Future perspectives and limitations

In this study, we investigated the effects of preferred music in both sexes. However, despite its importance, the menstrual cycle was not controlled in our experimental design. On the other hand, no female subject waited more than 72 hours to return to the laboratory to perform the second incremental test. Thus, although we cannot affirm that all female subjects performed tests restricted to the follicular or luteal phase, it is possible that huge variations of ovarian hormones in systemic circulation did not occur between tests.

Future studies are encouraged to investigate if our results can be transposed to other music characteristics (e.g., synchronous and asynchronous) or in other exercise types. Moreover, other physiological measurements during an incremental test, such as oxygen uptake and muscle oxygenation, can shed light on the effects of music during exercise.

## Conclusion

In summary, preferred music did not affect the iAT determination in an incremental running test, nor the physiological and perceptive responses at this intensity independently of sex. However, more than half of our female subjects had improved performance in the graded test with the preferred music, which may be more related to responses after iAT (severe domain) in this condition. These outcomes were not found for male subjects. Therefore, the effects of preferred music seem to be more pronounced for female subjects when compared to males.

## Supporting information

S1 File Parameters obtained from the incremental protocol performed in Trial 1 and Trial 2. (DOCX)

S2 File Table with descriptive data of the average and standard deviation, as well as percent in relation to the maximum score (i.e. 42 points), of each song score (BMRI-2) in their respective position in the playlist, as well as the mean value of the 10 songs. (DOCX)

S3 File Table with descriptive data of the average and standard deviation of each music tempo (bpm) in their respective position in the playlist, as well as the mean value of the 10 songs. (DOCX)

S4 File (XLSX)
