# Supplementary material for: Effects of preferred music on physiological responses, perceived exertion, and anaerobic threshold determination in an incremental running test on both sexes
Source: PLoS One. 2020 Aug 12;15(8):e0237310. doi: 10.1371/journal.pone.0237310 (PMC7423319; doi:10.1371/journal.pone.0237310)
Supplement: S1 File — (DOCX) [file pone.0237310.s001.docx]

**S1 File.** Parameters obtained from the incremental protocol performed in Trial 1 and Trial 2**.**

|  | **Male** | |  | | **Female** | |
| --- | --- | --- | --- | --- | --- | --- |
|  | **Trial 1** | **Trial 2** |  | **Trial 1** | | **Trial 2** |
|  | n(NM)=7 n(M)=3 | n(NM)=3 n(M)=7 |  | n(NM)=6 n (M)=4 | | n(NM)=4 n(M)=6 |
| **iAT (km.h^-1^)** | 11.5 ± 0.9 | 11.6 ± 1.2 |  | 9.6 ± 0.6 | | 9.9 ± 0.7 |
| **iAT (% i_peak_)** | 74.2 ± 3.4 | 73.4 ± 2.8 |  | 77.5 ± 6.1 | | 78.9 ± 3.4 |
| **[Lac]_iAT_ (mM)** | 3.7 ± 0.9 | 3.5 ± 0.8 |  | 4.5 ± 1.7 | | 5.2 ± 2.3 |
| **[Lac]_iAT_ (% [Lac]_peak_)** | 48.1 ± 6.3 | 45.9 ± 5.1 |  | 54.4 ± 13.7 | | 54.4 ± 11.5 |
| **HR_iAT_ (bpm)** | 152 ± 10 | 153 ± 10 |  | 164 ± 13 | | 165 ± 13 |
| **HR_iAT_ (%HR_max_)** | 76.9 ± 4.9 | 77.6 ± 4.8 |  | 81.8 ± 6.2 | | 82.7 ± 6.2 |
| **TT (s)** | 1663 ± 297 | 1691 ± 218 |  | 1089 ± 296 | | 1085 ± 233 |
| **TB_iAT_ (%TT)** | 59.5 ± 3.9 | 59.3 ± 5.6 |  | 59.3 ± 6.2 | | 61.7 ± 7.2 |
| **TA_iAT_ (%TT)** | 40.5 ± 3.9 | 40.7 ± 5.6 |  | 40.7 ± 6.2 | | 38.3 ± 7.2 |
| **RPE_Borg iAT_ (score)** | 13 ± 1 | 12 ± 1 |  | 13 ± 2 | | 13 ± 2 |
| **ETL_iAT_ (score)** | 12 ± 3 | 11 ± 3 |  | 12 ± 3 | | 12 ± 3 |

**iAT** − Anaerobic threshold intensity; **iAT (% i_peak_)**– Relativization of anaerobic threshold intensity in relation to the maximum intensity reached in protocol; [**Lac]_iAT_** – Blood lactate concentration at iAT; **[Lac]_iAT_ (% [Lac]_peak_)** ‒ Relativization of the lactacidemia referring to the iAT in relation to the lactate peak value obtained in the protocol; **HR_iAT_** – Heart rate at iAT; **HR_iAT_ (%HR_max_)** − Relativization of the heart rate referring to the iAT in relation to the product of the equation 220-age; **TT** – total time effort; **TB_iAT_ (%TT)** − Relativization of the time to reach the iAT in relation to the total time of effort; **TA_iAT_ (%TT)** − Relativization of the total time after reached the iAT in relation to the total time of effort; **RPE_Borg_ _iAT_** – rating of perceived exertion at iAT; **ETL_iAT_** – Estimation of time limit at iAT. ^γ^ significant difference between male and female in the preferred music condition; **NM** – Non-music condition; **M** – Music condition. ^†^ significant difference between male and female in the non-music condition. Significance was pre-fixed at p ≤ 0.05.
