# Supplementary material for: Effects of preferred music on physiological responses, perceived exertion, and anaerobic threshold determination in an incremental running test on both sexes
Source: PLoS One. 2020 Aug 12;15(8):e0237310. doi: 10.1371/journal.pone.0237310 (PMC7423319; doi:10.1371/journal.pone.0237310)
Supplement: S2 File — (DOCX) [file pone.0237310.s002.docx]

**S2 File.** Table with descriptive data of the average and standard deviation, as well as percent in relation to the maximum score (i.e. 42 points), of each song score (BMRI-2) in their respective position in the playlist, as well as the mean value of the 10 songs.

|  |  | **Music #1** | **Music #2** | **Music #3** | **Music #4** | **Music #5** | **Music #6** | **Music #7** | **Music #8** | **Music #9** | **Music #10** |  | **Mean±SD** |
| --- | --- | --- | --- | --- | --- | --- | --- | --- | --- | --- | --- | --- | --- |
| **Male** | **Score** | 42 ± 1 | 41 ± 1 | 41 ± 2 | 40 ± 1 | 39 ± 2 | 39 ± 2 | 38 ± 2 | 37 ± 3 | 36 ± 3 | 35 ± 4 |  | 39 ± 2 |
|  | **%** | 99.0 | 97.9 | 96.4 | 94.3 | 93.3 | 91.7 | 90.2 | 87.4 | 86.0 | 82.5 |  | 91.9 |
| **Female** | **Score** | 42 ± 0 | 42 ± 0 | 41 ± 1 | 40 ± 1 | 40 ± 1 | 40 ± 1 | 39 ± 1 | 38 ± 1 | 37 ± 2 | 34 ± 3 |  | 39 ± 1 |
|  | **%** | 99.8 | 99.3 | 97.6 | 96.2 | 94.3 | 94.0 | 91.9 | 90.7 | 87.1 | 81.4 |  | 93.2 |

**SD –** Standard deviation
