# Supplementary material for: Effects of preferred music on physiological responses, perceived exertion, and anaerobic threshold determination in an incremental running test on both sexes
Source: PLoS One. 2020 Aug 12;15(8):e0237310. doi: 10.1371/journal.pone.0237310 (PMC7423319; doi:10.1371/journal.pone.0237310)
Supplement: S3 File — (DOCX) [file pone.0237310.s003.docx]

**S3 File.** Table with descriptive data of the average and standard deviation of each music tempo (bpm) in their respective position in the playlist, as well as the mean value of the 10 songs.

|  |  | **Music #1** | **Music #2** | **Music #3** | **Music #4** | **Music #5** | **Music #6** | **Music #7** | **Music #8** | **Music #9** | **Music #10** |  | **Mean±SD** |
| --- | --- | --- | --- | --- | --- | --- | --- | --- | --- | --- | --- | --- | --- |
| **Male** | **bpm** | 128 ± 36 | 119 ± 20 | 119 ± 22 | 124 ± 21 | 128 ± 24 | 127 ± 29 | 113 ± 17 | 107 ± 20 | 116 ± 25 | 117 ± 24 |  | 120 ± 24 |
| **Female** | **bpm** | 126 ± 10 | 117 ± 22 | 130 ± 20 | 125 ± 20 | 123 ± 14 | 131 ± 17 | 123 ± 25 | 110 ± 23 | 122 ± 23 | 117 ± 18 |  | 122 ± 19 |

**SD –** Standard deviation
